# Cox’s Chair Revisited: Can Spinning Alter Mood States?

**DOI:** 10.3389/fpsyt.2013.00132

**Published:** 2013-10-15

**Authors:** Lotta Winter, M. Axel Wollmer, Jean Laurens, Dominik Straumann, Tillmann H. C. Kruger

**Affiliations:** ^1^Department of Psychiatry, Social Psychiatry and Psychotherapy, Division of Clinical Psychology, Medical School Hannover, Hannover, Germany; ^2^Asklepios Clinic North – Ochsenzoll, Asklepios Campus Hamburg, Medical Faculty, Semmelweis University, Hamburg, Germany; ^3^Department of Neurology, Zurich University Hospital, Zurich, Switzerland

**Keywords:** Cox’s chair, Hallaran’s swing, turntable, vestibular system, spinning, yaw stimulation, mood states, affective

## Abstract

Although there is clinical and historical evidence for a vivid relation between the vestibular and emotional systems, the neuroscientific underpinnings are poorly understood. The “spin doctors” of the nineteenth century used spinning chairs (e.g., Cox’s chair) to treat conditions of mania or elevated arousal. On the basis of a recent study on a hexapod motion-simulator, in this prototypic investigation we explore the impact of yaw stimulation on a spinning chair on mood states. Using a controlled experimental stimulation paradigm on a unique 3-D-turntable at the University of Zurich we included 11 healthy subjects and assessed parameters of mood states and autonomic nervous system activity. The Multidimensional Mood State Questionnaire and Visual Analog Scales (VAS) were used to assess changes of mood in response to a 100 s yaw stimulation. In addition heart rate was continuously monitored during the experiment. Subjects indicated feeling less “good,” “relaxed,” “comfortable,” and “calm” and reported an increased alertness after vestibular stimulation. However, there were no objective adverse effects of the stimulation. Accordingly, heart rate did not significantly differ in response to the stimulation. This is the first study in a highly controlled setting using the historical approach of stimulating the vestibular system to impact mood states. It demonstrates a specific interaction between the vestibular system and mood states and thereby supports recent experimental findings with a different stimulation technique. These results may inspire future research on the clinical potential of this method.

## Introduction

In 1804 Joseph Mason Cox published his book “Practical Observations on Insanity” (1). The chapter “swinging” gives a detailed description about how to construct a circulating chair and about benefits when this method was applied to psychiatric patients. Cox described: “one of its most valuable properties is its proving a mechanical anodyne. After a few circumvolutions, I have witnessed the soothing lulling effects, when the mind has become tranquilized, and the body quiescent; a degree of vertigo has often followed, and this been succeeded by the most refreshing slumbers” [(1), p. 104]. This kind of spinning intervention was mostly applied to patients suffering from mania, other forms of high agitation or catatonic stupors with the aim to calm them and put them into a milder condition (2). However, aversive side effects like “vertigo, attended by pallor, nausea, and vomiting; and frequently by the evacuation of the contents of the bladder” were common [(1), p. 106]. Critical retrospective reports about the application of the Cox’s chair in the beginning of the eighteenth century also document abusive applications in psychiatric patients for induction of behavioral change (3, 4). Apart from that Cox’s chair may have influenced investigations on vertigo, the construction of the Bárány chair for clinical assessment of vestibular function as well as funfair rides (3, 5).

Nevertheless, the idea of possible beneficial effects in the treatment of psychiatric patients has never been picked up by modern scientific investigations so far. Only recently, we have demonstrated that even brief and mild application of distinct vestibular stimulation on a motion-simulator (hexapod) may evoke significant alterations in mood states in healthy subjects (6). Specifically, we found that rotational movements may induce comforting and pacifying effects, whereas translational movements seem to evoke alertness.

Also from a clinical point of view there is evidence of a strong interaction between the vestibular system and mood states (7–9). For instance, there is a strong coincidence between vertigo and mental disorders, such as depression (10) and anxiety disorders (8, 11, 12). Further studies show that mood may influence the ability to keep one’s balance (13) and that depressed patients show side asymmetry in the activity of the vestibular nuclei assessed via the vestibulo-ocular reflex (14). Conversely, dysfunction of the vestibular system may trigger anxiety and depressive symptoms (8) and amelioration of associated mental symptoms has been documented after vestibular rehabilitation (15, 16) or after treatment with selective serotonin reuptake inhibitors (SSRI) (17). At the neuroanatomical level, there are projections from the vestibular nuclei via cerebellar, brainstem, and diencephalic centers to cortical and sub-cortical brain regions that are also involved in the regulation of mood states (18–22). These regions include the insula, the cingulate, the hippocampus, and the parabrachial nucleus. Moreover, the dorsal raphe and the locus coeruleus, two other important structures in the regulation of mood states, send out serotonergic and noradrenergic projections to vestibular nuclei in the brain stem (23, 24). But also on a psychophysiological level – although not yet empirically assessed – it can be observed that humans generally, and children in particular, seek movements that are associated with vestibular stimulation (such as swinging, dancing, sports) and may experience positive emotions.

These findings together with the recent results of our experiments on a hexapod motion-simulator led us to the idea of the “original spin doctors” (2, 4) and to explore if a vestibular stimulation may induce alterations of mood states. Healthy volunteers underwent a controlled moderate stimulation paradigm on the worldwide unique 3-D-turntable at the University Clinic in Zurich. In analogy to the historic specifications the vestibular system was stimulated by rotation around the yaw axis, i.e., the longitudinal body axis. Alterations of mood states were assessed by different psychometric instruments that proofed good reliability in our previous studies. As a measure of autonomic nervous system activity heart rate was continuously recorded.

## Materials and Methods

### Subjects

Eleven healthy volunteers [mean age of 28.84 ± 1.75 (SE); range 22–39 years] participated in this study after providing written informed consent. Four of them were male (mean age 31.78 ± 2.81; range 25–39) and seven were female [mean age 27.16 ± 2.1 (SE); range 22–38]. The subjects were recruited via advertisement on the Internet and on notice-boards at the University of Zurich, Switzerland. The advertisements included the information that the relationship between the equilibrium organ and mood states was investigated. The study was approved by the ethics committee of the canton Zurich, Switzerland.

In accordance with our previous study (6) all participants were screened by a semi-structured interview and by filling out a general socio-demographic and medical questionnaire. Individuals suffering from any somatic or mental disorders, depicting drug/alcohol abuse, or taking any kind of medication were excluded. We also assessed current or previous disorders of the vestibular system using the main items of the German version of the Vertigo Symptom Scale VSS-D (25, 26). Subjects with any signs of such disorders were excluded from participation.

Since the assessment of mood states was the principal aim of the current study, subjects were additionally asked to complete the Beck’s Depression Inventory [BDI, (27)] to exclude subjects with signs of a clinical depression. To control the factor that a propensity for sensation-seeking behavior may confound the rating of vestibular stimulation paradigms, we screened all participants with the Sensation-Seeking Scale (SSS) (28, 29). Both instrument, the BDI and the SSS, did not reveal clinically relevant scores in the participants.

### Design and procedure

We used a balanced cross-over design with an experimental and a control condition. Each subject had three appointments. The first one contained the examination of the inclusion and exclusion criteria and a test stimulation for the subject for habituate to the turntable. The second and third appointments comprised the actual investigation sessions in a randomized order.

**Figure 1:**
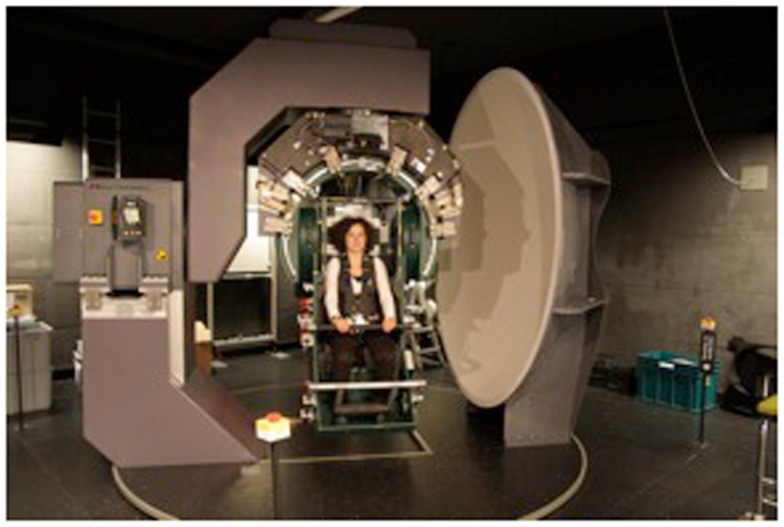
**The 3-D-Turntable**. A 3-D-Turntable is a mechanical device, which operates as a three-axis stimulator. It can be driven into any position in space and turn in any angle desired up to 360°.

The vestibular stimulation was performed on a 3-D-turntable (Figure 1). As described previously (30) the three-axis rotational stimulator is driven by three servo-controlled motorized axes (Acutronic, Switzerland), controlled with Acutrol software and hardware, and interfaced with Lab VIEW software. Subjects are comfortably seated in a chair and secured with safety belts. The center of the head is positioned at the center of the rotation. Individually adjusted masks (Simmed BV, Reeuwijk, The Netherlands), made of a thermoplastic material (Posicast), were molded to the contour of the head after warming with an opening in the mask made for the mouth. The mask is attached to the back of the chair, and restricts head movements very effectively without causing discomfort (30).

The vestibular stimulation consisted in rotating the subject around an earth-vertical axis at a velocity of 396°/s. The rotation started with an acceleration at 66°/s^2^ and lasted for 6 s. It was followed by a 100 s of constant-velocity rotation. The rotation was ended by a gentle deceleration (15 s at 26.4°/s^2^). The control condition consisted in a similar rotation profile, but at a much lower velocity which was below the detection threshold of the vestibular organs. However, the level of noise production of the turntable was similar in each session, so that subjects were not able to distinguish between control and experimental session regarding the environmental factors. The order of experimental and control session was randomized. To minimize possible effects of circadian rhythms on variance in mood states and heart rate, all sessions took place between 2 and 8 p.m., with both control and experimental sessions for each subject taking place at the same time. Before and after the stimulation subjects were asked to complete psychometric instruments. In addition the plot of each participant’s heart rate was measured to detect changes of cardiovascular activity as an indicator of autonomous nervous system activity.

### Measures

#### Psychometric measures

As in our previous study (6) for measurements of mood states we used five 100 mm bipolar visual analog scales (VAS, ranging from 0 to 100 with 50 representing “neutral”). The five VAS were “good – bad,” “energized – exhausted,” “relaxed – tense,” “comfortable – uncomfortable,” and “alert – sleepy.” VAS are sensitive and valid tools for the immediate assessment of mood and psychophysiological alterations (31, 32). Furthermore, the original German short version of the Multidimensional Mood State Questionnaire (MDBF) was used (33). The MDBF is a self-reporting instrument to measure the current psychological and mood state in three global dimensions (“good mood – bad mood,” “alertness – tiredness,” and “calmness – agitation”). The questionnaire consists of 12 items in the short version and includes 5 point Likert scales (1–5). Four items constitute one global dimension or subscale. The scores of a subscale range from 4 to 20 with higher values representing the positive pole and lower values the negative pole of a mood state. After each stimulation participants were asked to rate how they felt during the stimulation by completing the five visual analog scales as well as the MDBF.

We also asked participants for brief free text comments and descriptions of associations they had after the stimulation paradigm.

#### Cardiovascular measures

As a measurement of autonomous nervous system activity, heart rate was continuously recorded using the Polar RS 800 Multi (Polar Electro Oy, Finland). This device includes a wristwatch that serves as a receiving and storing device for the data sent by wireless transmission from a chest strap. The heart rate was measured beat to beat. The heart beat curve was also plotted by using an interval of 5 s starting 10 s before the stimulation began.

### Statistical analysis

As deviations from normal distributions could not be proven for all psychometric data the non-parametric Wilcoxon-Test was used to compare medians in a pre-post comparison of both the experimental and the control condition. Additionally, to classify how relevant the results of the comparison of medians are, corrected effect sizes (Cohen’s d) were calculated. According to Cohen a *d* ≥ 0.2 is considered as a small, *d* ≥ 0.5 as a medium, and *d* ≥ 0.8 as a large effect size (34). For the cardiovascular data normal distribution could be verified. These data were analyzed by a two-way analysis of variance (ANOVA, condition × time) for repeated measures. Data were analyzed using PASW 18.0 (SPSS Inc., Chicago, IL, USA). An α of 0.05 was considered statistically significant for all analyses.

## Results

### Psychometric measures

**Figure 2:**
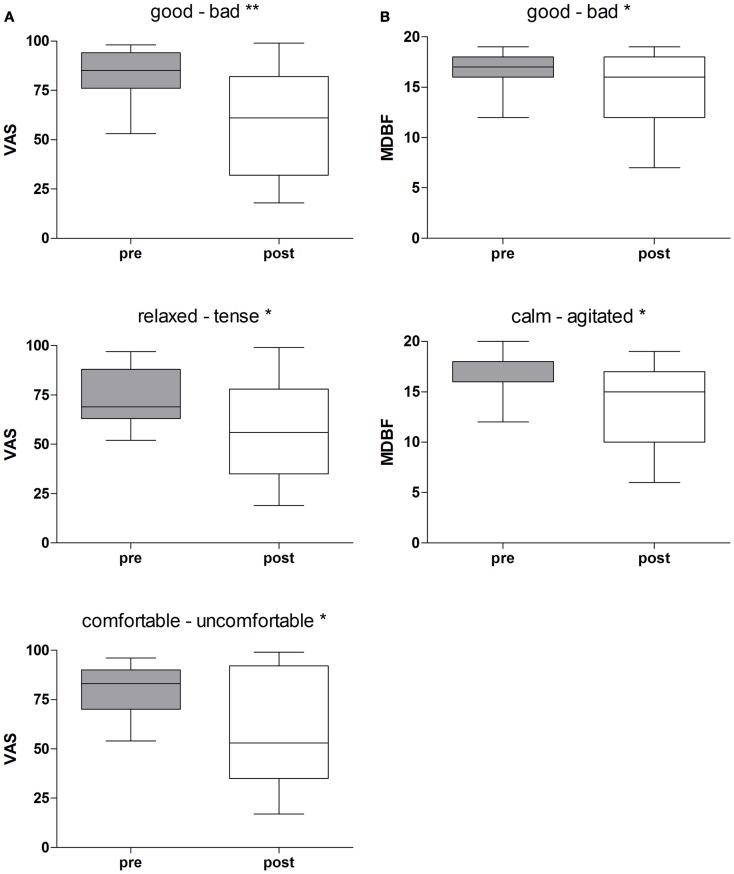
**(A)** Rotational stimulation and impact on mood states using VAS-scales. Boxplots of significant effects of the rotation on mood states using VAS-scales in a pre-post comparison. Boxplots showing the median, upper, and lower quartile, with whiskers showing the minimum and maximum of all values. High values on the VAS indicated that the positive adjective of the pair was appropriate and vice versa for low values. *p < 0.05, **p < 0.01. **(B)** Rotational stimulation and impact on mood states using MDBF-scales. Boxplots of significant effects of the rotational stimulation on mood states using MDBF-scales in a pre-post comparison. Box plots showing the median, upper, and lower quartile, with whiskers showing the minimum and maximum of all values. High values indicated that the positive adjective of the pair was appropriate and vice versa for low values. *p < 0.05.

Rotational yaw stimulation on a turntable (spinning stimulation) induced specific changes on mood states as measured with the visual analog scales (VAS) and the MDBF. In a pre-post comparison the VAS-scales indicated that subjects felt less “good” (*Z* = −2.58, *p* = 0.01), less “relaxed” (*Z* = −2.05, *p* = 0.04), and less “comfortable” (*Z* = −2.31, *p* = 0.02) after yaw stimulation (Table 1; Figure 2A). The outcome of the MDBF scales support these tendencies as the subjects stated to feel “bad” (*Z* = −2.14, *p* = 0.03) and “agitated” (*Z* = −2.06, *p* = 0.04) (Table 1; Figure 2B). Per contra, looking at the subjective additional notes only one subject described a “pretty bad feeling.” Other validations quoted comparisons with funfair rides and said it was fun. In contrast, the control session did not induce any significant alterations of mood states (Table 1).

**Table 1:** **Results of statistical analysis of the (A) VAS and (B) MDBF-scales**.

		Pre				Post				Wilcoxon		ES
		*M*	Med	SD	*N*	*M*	Med	SD	*N*	*Z*	*p*	*d*_corr_
**VAS**												
Good – bad	E	81.91	85	14.495	11	58.36	61	27.725	11	−2.584	**0.01**	**0.83**
	C	78.82	81	14.764	11	72.73	85	17.124	11	−1.469	0.142	
Energized – exhausted	E	71.45	79	20.786	11	69.00	62	25.534	11	−0.445	0.657	0.02
	C	69.91	73	16.183	11	66.55	79	21.690	11	−0.510	0.610	
Relaxed – tense	E	74.64	69	14.975	11	58.55	56	25.828	11	−2.045	**0.041**	**0.91**
	C	74.18	78	17.526	11	77.82	69	17.244	11	−1.026	0.305	
Comfortable – uncomfortable	E	79.36	83	13.056	11	57.18	53	30.436	11	−2.312	**0.021**	**0.96**
	C	76.00	84	18.531	11	75.73	83	17.664	11	−1.123	0.261	
Alert – sleepy	E	71.82	71	20.990	11	75.27	82	24.422	11	−0.802	0.423	**0.41**
	C	70.36	70	19.459	11	65.00	71	17.349	11	−1.224	0.221	
**MDBF**												
Alert – sleepy	E	14.36	15	2.838	11	14.64	16	3.501	11	−522	0.602	0.09
	C	13.82	14	3.459	11	13.82	14	2.857	11	−0.512	0.609	
Good – bad	E	16.45	17	2.252	11	14.55	16	3.857	11	−2.142	**0.032**	**1.24**
	C	15	16	3.821	11	17.18	17	1.662	11	−1.492	0.136	
Calm – agitated	E	16.73	18	2.370	11	14.09	15	4.277	11	−2.057	**0.040**	**1.27**
	C	16	17	3.130	11	17.36	17	1.629	11	−1.340	0.180	

As the normal distribution could not be assumed, the non-parametric Wilcoxon-Test was executed as well as corrected effect sizes were calculated to value the effects. Effect size (ES), Mean (M), Median (Med), Standard Deviation (SD), sample size (N), corrected effect size (*d*_corr_). Significant results are highlighted in bold font.

We calculated effect sizes considering an interaction among the conditions and time, or allowing for development among the three groups. As such, three large effect sizes were found on the scales “good – bad” (*d*_corr_ = 0.83), “relaxed-tense” (*d*_corr_ = 0.91), and “comfortable-uncomfortable” (*d*_corr_ = 0.96) of the VAS-scales (Table 1). Additionally, one small effect size appeared with the scale “alert-sleepy” (*d*_corr_ = 0.41). The arithmetic mean shows that during the control condition the subjects’ alertness faded whereas in the experimental session alertness increased. The results of the MDBF scales show large effect sizes on the scale “good – bad” (*d*_corr_ = 1.24) and on the scale “calm – agitated” (*d*_corr_ = 1.27) (see Table 1).

### Cardiovascular measures

The ANOVA did not reveal any significant differences in heart rate between the experimental and the control condition. Ten seconds before start of stimulation mean heart rate was 76.4 bpm in the control and 76.1 bpm in the experimental session. There were no significant alterations of heart rate over time during both conditions (data not shown).

## Discussion

Everyday life experience, clinical observations and historical aspects indicate a close interaction between the vestibular system and mood states. However, experimental studies on this subject are missing and the present investigation may provide an early contribution to close this gap. We observed that even a brief spinning stimulation on a 3-D-turntable is able to significantly alter mood in healthy subjects. In contrast to our previous study on a hexapod motion-simulator the current stimulation on a turntable predominantly downgraded parameters of mood in terms of feeling less good, less relaxed, and less comfortable, whereas alertness increased. One might argue that aversive aspects of the experimental setting, namely head and body fixation on a technical device in a dark room, may have contributed to this mood changes. However, the aversive aspects were also present in the control condition in which the described mood changes did not occur. Thus, the mood changes were specifically related to vestibular stimulation. At the same time none of the parameters reached median values below 50 for the VAS or below 12 for the MDBF, which would correspond to the negative section of the bipolar mood scales. Therefore, the paradigm may have rather mood-allaying or equalizing effects. Such mechanisms may have contributed to the effects observed in the ancient use of Cox’s chair in the treatment of psychiatric patients in states of arousal (3, 5).

The lack of increase in the heart rate as a parameter of sympathetic nervous system activation and surrogate marker of stress is in line with the absence of strong negative emotions like fear during the experiment. Some participants actually enjoyed the experiment and compared it to a funfair ride, which may correspond to historical anecdotal reports that some patients used the circulating swing as a source of entertainment (2).

The current investigation only included healthy subjects without signs of any mood disorder. Whereas in such studies ceiling effects may diminish results we were still able to detect significant changes in mood states with considerable effect sizes. Future studies in this field may want to include subjects with affective disorders such as depression or mania for further exploring the theory of mood balancing effects. In this context an early observation by the German psychiatrist Emil Kraepelin is of interest (35) saying that “healthy people begged for the machine to be stopped before 2 min had passed whereas mental patients endured the experience for as long as 4 min.” Hallaran goes as far as to state that the circulating swing was also a source of entertainment for some patients (2). Thus, even the vestibular system – which includes various neuronal structures, albeit barely focused by psychiatric research yet – may be substantially affected by mental processes and vice versa. This is supported by recent findings of an altered vestibular function in subjects with mental disorders as described by Soza Ried and Aviles (14) who found hypoactive vestibular nuclei and functional side asymmetry in major depression as assessed by measurements of the vestibulo-ocular reflex (14). Consequently, the question arises whether vestibular stimulation can also be used to restore such dysfunctions.

Some limitations of the study should be addressed. The sample size (*n* = 11) was small. Moreover, the experiment comprised only one experimental condition. It is possible or even probable, that different amplitudes, frequencies, durations, planes, and axes of rotation may produce differential mood effects. The experiment was carried out only once. It is possible that, either by cumulative effects or by adaptation, repetitive, or serial stimulation over a longer period of time may produce other mood effects. The experiment was carried out in the dark. It is possible that under condition allowing for visual adjustment, mood effects may turn out in a different way. Finally, the current approach incorporated only subjective psychometric measures together with heart rate which could be complemented by additional objective neurophysiological measures in future studies to achieve additional information on related cerebral function. All these issues may be addressed in future studies on the interaction of vestibular stimulation and mood. The effect of spinning is not restricted to vestibular stimulation but also produces visceral acceleration. We cannot exclude, that the corresponding sensory input may have contributed to the observed mood effects.

Thankfully, the use of Cox’s chair, which was also associated with highly aversive side effects and had been applied abusively in psychiatric history, was abandoned. With a moderate and well tolerable experimental paradigm we have recapitulated aspects of this ancient device in healthy volunteers. Our findings indicate that spinning, possibly mediated by its effects on the vestibular system can alter mood states. This implies the possibility that stimulation of the vestibular system, be it caloric, galvanic, or movement-induced, may be a therapeutic access to the modulation of mood states. This approach is supported by findings of an altered vestibular function in subjects with psychiatric disorders as mentioned above (14). Phobic conditions, for instance, are frequently associated with dizziness and there is evidence that vestibular training may contribute to clinical improvement (11, 36, 37).

## Conclusion

This prototypic investigation provides first evidence that vestibular stimulation on a 3-D spinning chair has a specific impact on mood states and may be used for studying the well-known, although barely studied, link between the vestibular system and the emotional brain. This may inspire further research into this connection and its therapeutic potential.

## Conflict of Interest Statement

The authors declare that the research was conducted in the absence of any commercial or financial relationships that could be construed as a potential conflict of interest.
